# Mālama nā makua i nā keiki me ka hānō: Native Hawaiian Parents Caring for Their Children with Asthma (Part 2)

**DOI:** 10.31372/20190403.1020

**Published:** 2019

**Authors:** May K. Kealoha, Sandra L. Sinclair, Karol K. Richardson

**Affiliations:** a Kapi‘olani Community College, Honolulu, HI 96816, USA; b University of Hawai‘i Mānoa, Honolulu, HI 96822, USA

**Keywords:** native Hawaiian, culture, indigenous, childhood asthma, uncertainty, focus group, parents, rural, geographic barriers

## Abstract

*Objective*: Native Hawaiian children have the highest prevalence of asthma among all ethnicities in Hawai‘i. Mālama Part 2 describes continuing research, exploring contemporary native Hawaiian parents’ perspective, and experience of caring for their children with asthma in the context of uncertainty while living on the islands of Hawai‘i, Kaua‘i, Maui, Moloka‘i and Lāna‘i. *Design*: Descriptive qualitative approach by means of directed content analysis using focus groups was applied to this study. Eight open-ended questions elicited asthma history, asthma management, and how the Hawaiian culture affects parents’ health practices. Directed content analysis applied Mishel’s Uncertainty in Illness Theory (UIT) to guide data collection, organization, and analysis. *Sample*: Thirty-three native Hawaiian parents with a child with asthma met in 9 separate focus groups during 2012–2015 on the islands of Hawai‘i, Kaua‘i, Maui, Moloka‘i, and Lāna‘i. *Results*: The study’s findings were congruent with the first Mālama study results of focus groups on O‘ahu. Contextual influences including indigenous worldview, cultural values, history, and assimilation and acculturation factors affected native Hawaiian parents’ perceptions and experiences with conventional asthma care. Moreover, Hawaiian parents living on islands outside of metropolitan O‘ahu reported geographic barriers that contributed to their uncertainty. *Conclusion*: Political action is required for comprehensive medical care, health education, and nursing services to be delivered to families living on all islands. Integrating Hawaiian cultural values, involving ‘*ohana*, and applying complementary alternative therapies as well as standard asthma management will strongly support native Hawaiian parents caring for their children with asthma.

## Introduction

Native Hawaiian children have the highest prevalence rate of asthma among children of all ethnicities in the State of Hawai‘i (DOH, 2015). In a first component of the qualitative study, “*Mālama nā makua i nā keiki me ka hānō: Native Hawaiian parents caring for their children with asthma*” (Kealoha & Kataoka-Yahiro, 2017), it was noted that native Hawaiian parents’ indigenous worldview, cultural values, historical context, and effects of assimilation and acculturation contribute to an experience of uncertainty regarding conventional asthma care. This article describes continued *Mālama* research regarding the perceptions and experiences of native Hawaiian parents caring for their children with asthma in the context of uncertainty while living on the islands of Hawai‘i, Kaua‘i, Maui, Moloka‘i, and Lāna‘i in *ka pae ‘āina* (Hawaiian archipelago), whereas the first component of the research focused on families living on the island of O‘ahu.

## Background

Asthma is one of the most prevalent chronic childhood diseases in the United States (Horner, Brown, & Walker, 2012). Nationally, 6.2 million children currently have asthma, representing 8.4% of children in the United States (CDC, 2015). Asthma is a major public health concern for the state (DOH, 2014a). Child asthma status for Hawai‘i for years 2012 through 2015 ranged from 9.9% to 16% of the population (DOH, 2012, 2013, 2014b, 2015). According to the Hawai‘i School Health Survey, a high prevalence of asthma among youths (grades 9^th^–12^th^) with current asthma was reported in every county in 2015: Hawai‘i (15.8%), Honolulu (12.4%), Kauai (16.6%), Maui (12.9% includes Moloka‘i and Lāna‘i) (Hawaii Health Data Warehouse, 2017).

An ethnicity-related disparity among children with asthma exists. Native Hawaiian children have significantly higher asthma prevalence (19.4%) compared to the percentage of children of other ethnicities (White 7.1%, Filipino 7.3%, Others 7.3%) (DOH, 2015). The disparity is evident for native Hawaiian youths living in each county for 2015: Hawai‘i (20.9%), Honolulu (12.4%), Kauai (22.2%), and Maui (19.8% includes Moloka‘i and Lāna‘i) (Hawaii Health Data Warehouse, 2017). See Table 1.

**Table 1:** Current Youth Asthma Status by County and Race, Years 2013 and 2015

Race – Ethnicity	Hawai‘i		Honolulu		Kaua‘i		Maui	
Year	2013	2015	2013	2015	2013	2015	2013	2015
All ethnicities combined	16.6%	15.8%	11.9%	12.4%	14.3%	16.6%	12.5%	12.9%
Caucasian	n/r	n/r	n/r	12.8%	n/r	8.8%	n/r	8.2%
Native Hawaiian	19.8%	20.9%	19.1%	12.4%	15.6%	22.2%	17.6%	19.8%
Filipino	21.1%	n/r	7.1%	11.7%	14.2%	16.1%	8.3%	10.4%
Japanese	n/r	n/r	n/r	9.9%	n/r	n/r	n/r	n/r
Black	n/r	n/r	n/r	n/r	n/r	n/r	n/r	n/r
Other Asian	n/r	n/r	n/r	6.0%	n/r	n/r	n/r	n/r
Other	14.3%	14.2%	14.7%	15.5%	15%	21.3%	14.2%	12.7%

*Note*: Adapted from Lifetime and Current Asthma in Hawaii, by County. The Hawaii Health Data Warehouse; State of Hawaii, Hawaii School Health Survey, Youth Risk Behavior Survey Module. Retrieved: http://hhdw.org/wp-content/uploads/C_YRBS_Chronic-Diseases_IND_00001C.pdf. 2014 data unavailable. Youth: High School Grades 9^th^–12^th^; n/r: not reportable.

Research regarding native Hawaiian parents and their care of children with asthma is sparse, and particularly research concerning families living on the rural islands of the state. Tse and Palakiko (2004) found that native Hawaiian parents living on O‘ahu did not recognize asthma symptoms, were unaware of the seriousness of the condition, and relied heavily on rescue asthma medications and their physician.

### Statement of Problem & Research Question

This study explores contemporary native Hawaiian parents’ perspective and experience caring for their children with asthma in the context of uncertainty. The initial research involved native Hawaiian parents living on O‘ahu. The research continued to include native Hawaiian parents living on the islands of Hawai‘i, Kaua‘i, Maui, Moloka‘i and Lāna‘i by incorporating the identical research question, design, recruitment, data collection, sample, data analysis, and assurance of rigor. Indigenous people should have a voice in the development of asthma programs and be given opportunities to express what types of support and assistance would be of true benefit to them. Contextual influences including worldview, cultural values, history, and assimilation and acculturation factors may affect native Hawaiian parents’ perceptions and experiences with conventional asthma care.

#### Research Question:

What are native Hawaiian parents’ perspective and experience of caring for their children with asthma in the context of uncertainty?

## Methodology

### Design

The methodology described in *Mālama nā makua i nā keiki me ka hānō: Native Hawaiian parents caring for their children with asthma* (Kealoha & Kataoka-Yahiro, 2017) is repeated here. Descriptive qualitative approach by means of directed content analysis using focus groups was applied to this study. A theoretical guide for data collection and analysis was utilized to ensure appropriate organization, collection of relevant data, and interpretation of findings (Canino et al., 2009). Mishel’s Uncertainty in Illness Theory (UIT), a well-established nursing theory based on the concept of uncertainty, served as the study’s theoretical framework (Mishel, 1990). The framework was broad and able to incorporate contextual features such as indigenous culture, cultural practices, and health beliefs (Walker & Avant, 2011). The UIT specifically contained constructs and categories that concentrated on the antecedents of uncertainty, modulating factors that increased or decreased uncertainty, and described attributes and implications of uncertainty (Mishel, 1990). A comprehensive assessment and understanding of parents’ uncertainty experience was completed by utilizing the UIT.

Focus groups for data collection in this study allowed native Hawaiian parents to respond to open-ended questions in their own words and from their own unique perspectives regarding health, beliefs, values, practices, cultural interpretations, and cultural insights (Bernard & Ryan, 2010; Castleden et al., 2016). The focus groups were conducted in the informal “talk story” format that was an acceptable form of gaining information from native Hawaiians (Andrade, 2008). The focus group questions adhered to three major guidelines: (1) respect cultural etiquette and practice of asking questions, sharing information, and applying Hawaiian words and concepts (Andrade, 2008); (2) apply focus group process of asking broad questions followed by more specific questions (Krueger & Casey, 2009); (3) align with the constructs of the UIT (Mishel, 1990). The initial questions were reviewed and revised by three native Hawaiian consultants familiar with asthma management. A pilot focus group was conducted with one group of parents meeting the inclusion criteria. The final eight open-ended questions are as follows:

1. When did it (illness/asthma) start?
2. What do you do to care for your child with asthma?
3. What things or people have made it easier for you as a parent caring for your child with asthma?
4. What things have made it harder for you as a parent?
5. What is it like for you as a parent taking care of your child?
6. How do you manage caring for your child with asthma?
7. How has your Hawaiian culture influenced you in the care of your child?
8. What matters most to you as a parent about this illness or treatment?

### Sample

A purposive sample of native Hawaiian parents with children with asthma was recruited with no restrictions regarding age, socioeconomic status, gender, or number of children to obtain a wide range of perspectives. The inclusion criteria of eligible parent participants were as follows: (a) self-reports as of Hawaiian ancestry; (b) is a biologic parent; (c) over 18 years of age; (d) self-reports no cognitive or emotional impairment; (e) has a child’s age less than 18 years of age; and (f) reports child has had current asthma for one year or more. As there may be multiple parental roles in families, biological parents were specifically recruited. The asthma history of one year or more confirmed the requisite for parents being exposed to the illness.

### Recruitment

Recruitment of participants on the islands involved recruiting *key informants* to identify prospective participants and linking them with the researcher (Krueger & Casey, 2009). There were a total of 33 participants; nine focus group sessions ensued. See Table 2. Each participant was offered incentives of $50 and two bags of poi (pounded cooked taro), a highly valued cultural food. The settings were at community centers, public library, school classrooms, health center, and an informant’s home. The sites were selected for convenience, accessibility, freedom from interruptions, and a sense of neutrality for participants.

**Table 2:** Focus Groups, Number, and Gender of Participants

Group	1	2	3	4	5	6	7	8	9	Total
Island	Lāna‘i	Lāna‘i	Hawai‘i	Hawai‘i	Kaua‘i	Kaua‘i	Kaua‘i	Molo-ka‘i	Maui	
Mother	2	2	4	5	2	1	1	5	3	25 (76%)
Father	2	1	1	1	1	1	1	0	0	8 (24%)
Subtotal	4	3	5	6	3	2	2	5	3	
Grand Total										33 (100%)

### Data Collection

The research team consisted of a principal investigator and research assistant. Data sources included a demographic survey and focus group discussions. Data collection period was from August 2012 to April 2015. Prior official approval was obtained from the University of Hawai‘i at Mānoa, Human Studies Program (CHS#19503). A protocol or focus group procedure was implemented for each focus group to maintain consistency and order; the procedure ensured participant attendance and complete set-up of the physical facility. Informed consent was obtained prior to the start of the session, and 2 hours were allotted for each focus group.

### Data Analysis

Data analysis included descriptive analysis of the demographic data and direct content analysis of the focus group sessions. Data from the focus group sessions were collected from three sources: (a) individual participants’ responses; (b) responses that occurred due to the interaction of the participants as they heard and responded to each other; and (c) consensual and differing group opinions (Houser, 2012; Krueger & Casey, 2009). Through directed content analysis, data were investigated in order to understand, digest, synthesize, conceptualize, and re-conceptualize descriptions of feelings, behaviors, experiences, and ideas (Hsieh & Shannon, 2005; Olsson, Eriksson, & Anderzén-Carlsson, 2017; Presseau et al., 2017). Subsequently, interpretive coding of the data was conducted to identify constructs, categories, subcategories, and new themes (Houser, 2012; Presseau et al., 2017).

Codes of constructs, categories, and subcategories, and their relationships were predetermined by the UIT (Hsieh & Shannon, 2005; Olsson et al., 2017; Presseau et al., 2017). UIT constructs consisted of the Stimuli Frame, Cognitive Capacities, Structure Providers, Uncertainty, Appraisal, and Adaptation. Categories were branches or off-shoots of constructs. Subcategories became more specific dimensions of categories. If the UIT directed codes of constructs, categories, and subcategories did not reflect data, then alternative codes were created to identify new themes (Hsieh & Shannon, 2005; Presseau et al., 2017).

The principal investigator was present during each focus group meeting. The research assistant had available audiotapes and transcriptions of each session. Content analysis was first performed by the research team members manually and then data was entered into NVivo (NVivo, 2011).

Through the process of construct and category identification and comparison of data, key ideas that frequently reflected the meaning of the data were established (Burns & Grove, 2011). Eventually, information appeared redundant with no new information being gained. Each UIT construct, category, and subcategory contained applicable coded data. After Focus Group 9 transcripts were reviewed and coded, the research team, including the external reviewer, determined by consensus that data saturation had been achieved and data collection ceased.

### Assurance of Rigor

Rigor was ensured by maintaining a strict process of data collection and analysis. Aspects of rigor in qualitative research included trustworthiness of the data collected (initial testing of questions by native Hawaiian consultants, validating findings with participants), confirmability (consistency and ability to duplicate decision making regarding data collection and data analysis were enhanced through the use of NVivo), and credibility of the findings (having summaries reviewed by participants through “member checks”) (Houser, 2012). Reliability of the findings was assured through internal consistency and inter-rater reliability of the coding system by the research team members as well as an external check that was completed by an external reviewer who independently analyzed the data (Bernard & Ryan, 2010).

## Results

### Background Setting

The islands of O‘ahu, Maui, and Kaua‘i are similar in size, ranging between 550 and 780 square miles, with populations of 976,000, 148,000, and 69,000, respectively. Hawai‘i is the largest island consisting of 4,000 square miles and a population of 189,000. The land area of Moloka‘i is 260 square miles and it has a population of 7,000. Lāna‘i is the smallest island consisting of 141 square miles and a population of 3,500 (State of Hawaii Data Book, 2015).

### Demographic Characteristics

There were 9 focus groups with a total of 33 participants: 25 (76%) females and 8 (24%) males. See Table 2. The majority of participants were single (46%), but the marital status of 5 (15%) is unknown. Most parents had completed high school and 46% of parents had greater than high school education. The ages of the parents ranged from 19 to 54, with a mean age of 35 years. Most parents (70%) were employed. All the children had medical insurance and an identified health care provider. Medicaid was a common insurance provider (52%). Eight (24%) children had private health insurance, but the insurance provider for 8 (24%) children was unspecified.

Each parent was asked to answer questions based on one child with asthma (total of 33 children). Most of the children were male (58%). Parents reported more often on their oldest child (30%) or youngest child (36%). Children’s age groups included 2 (6%) toddlers, 5 (15%) preschool age, 19 (58%) elementary students, 5 (15%) middle school students, and 2 (6%) high school students. Four (12%) children were the only child in the family with asthma. Most children (52%) had one sibling with asthma.

### Uncertainty in Illness Theory

The study findings indicated that native Hawaiian parents experience uncertainty while caring for their child with asthma. Each UIT construct, category, and subcategory was individually addressed and supported by significant statements and quotes made by the participants. The four new subcategories (*Asthma Experience*, *Asthma Triggers*, *Direct Action-Western*, and *Complementary and Alternative Medicine*) that emerged from the O‘ahu study results were repeated in this study.

### Construct: Stimuli Frame, Category: Event Familiarity, Subcategory Asthma Experience

The *Asthma Experience* subcategory identified parents with limited asthma experience as having more uncertainty than parents with many years of asthma management. A parent who had recently resumed care of her child expressed unfamiliarity with asthma care. A well-educated parent felt very uncertain about asthma care. “I’ve not gotten any education about asthma through the doctors. None whatsoever. All they do is say, ‘Here you go. Take this (medication).’ And on your merry way.”

### Construct: Stimuli Frame, Category: Event Familiarity, Subcategory Asthma Triggers

*Asthma Triggers* was identified as a subcategory because unidentified triggers and unpredictable effects of asthma triggers were strong antecedents to uncertainty for parents. Parents specified asthma triggers common to their locale. Wet and rainy areas of Kaua‘i and Hawai‘i fostered the outgrowth of persistent mold on the walls of homes and in the ground. Areas in Maui are humid and moist with poor ventilation in older homes. One parent noticed fewer cockroaches in dry areas of Maui. Parents living on Lāna‘i and Moloka‘i complained about pollen and the persistent dust due to winds over agricultural lands. “Vog [volcanic air pollution] makes asthma worse” was repeated by parents on each island.

### Construct: Cognitive Capacities

Participants did not report any difficulty processing information about asthma care. Two single fathers, however, reported being thankful that they were able to rely on members of their ‘*ohana* (family) to implement asthma treatment for their child.

### Construct: Structure Providers, Category: Social Support

Major positive structure providers were health care providers, including pediatricians, primary care physicians, and ‘*ohana*. A few parents mentioned seeking treatment from a naturopath or chiropractor because they were dissatisfied with medical doctors and wanted “more natural” treatments. One parent described her difficult experience with a specialist.

I have yet to experience a good physician…. I did go to O‘ahu…took her to a specialist and it didn’t go well…. I was hoping to get good care for her. And when I went to the appointment…. I felt like, he talk[ed] down to me, and he was talking at me and not to me…. They said he’s a good physician but I felt that he had the worse bedside manner ever…. I scheduled another appointment, and I did not ever go back…. It’s a lot…to go off island and take your child…To get airfare…car… in one day…catch the flight back in the same day. What if you go and that doctor doesn’t work out?…So I pretty much just see the doctor here. He’s not a specialist and just to get her medication that she needs. But I feel that it’s not the best care…. I still need to put in place…emergency action plan for her.

‘*Ohana* served as the participants’ primary social support. The ‘*ohana* assisted parents in decreasing their uncertainties about asthma symptoms and management by providing information, service-related assistance (child care, meal preparation, medication administration, transporting child to doctor’s office), and emotional support. ‘*Ohana* consisted of spouses, mothers, fathers, sisters, grandparents, aunts, uncles, cousins, mothers-in-law, fathers-in-law, sisters-in-law, brothers-in-law, godparents, girlfriends, and boyfriends. Fathers were actively involved with their children’s asthma management by administering medication, communicating with the physicians and school personnel, and minimizing children’s exposure to asthma triggers. Parents living on smaller islands considered “everyone” in the community to be ‘*ohana*.

A few parents lacked spousal assistance due to marital separation. Other parents reported not having ‘*ohana* assistance because members lived on different islands. Single parents with more than one child exhibiting asthma symptoms at the same time experienced the most difficulty. Securing competent babysitters posed a challenge for parents who worked.

### Construct: Uncertainty, Lack of Information

Lack of information contributed to the uncertainty experience of parents. Questions were raised about the etiology of asthma because there are “too many kids (with asthma).” What are the causes of asthma? Is there a genetic link? What about the environment? Some parents wondered, “What did I do?”

### Construct: Appraisal: Inference of Danger, Category: Coping Mobilizing Strategies, Direct Action – Western, Complementary and Alternative Medicine (CAM)

UIT coping strategies included four subcategories also identified by the research team in the previous report: vigilance, information seeking, direct action – Western, and complementary and alternative medicine (CAM). Parents were vigilant in monitoring for asthma symptoms and asthma triggers. One focus group recommended having asthma information provided to school classmates to further vigilance. “Now, you have more eyes than just*…*parents.”

Participants sought informational support about asthma from health professionals, ‘*ohana*, internet, Child Family Services, Kaiser Permanente asthma classes for parents and children, and the American Lung Association. Many participants asked for support groups and education classes with updated information for children, parents, families, friends, school personnel, teachers, and community. Here is one parent’s recommendation.

I think they should get one program where they can go to every town cause not everybody get cars, not everybody fortunate to send their kids here and there… Would be nice… And that way the people from that community can show up, they don’t have to travel far… I know we get the buses. Sometimes you catch the bus but you still have to walk miles to get to where eva [wherever]…

Although the participants did not specifically label prescribed medical treatment as “Western” therapy, they appeared to recognize medical care by “doctors” versus the “Hawaiian way.” All participants, except one, reported administering prescribed medical treatment (medication, aerosol treatment) for effective relief from respiratory distress.

Parents offered three types of CAM therapies that were identified and categorized by the research team: (a) comfort measures, (b) home remedies, and (c) Hawaiian cultural healing practices. Comfort measures included having the child stop running, encouraging sitting and relaxing, persuading them to breathe deeply and calmly, and refocusing the child’s attention. Home remedies were “handed down” by parents’ own mothers or *kūpuna* (family elders) and included applying Vicks to one’s chest, back, and/or underarms; warm baths, hot showers and inhaling steam of water, Vicks, or vinegar; drinking hot water, warm tea, or tea with ginger; and drinking caffeinated drinks such as “hot black coffee.” One group reported the effectiveness of “dōTERRA essential oils” with massage.

Hawaiian cultural healing practices were taught to participants by parents, grandparents, extended family members, friends, and/or healing practitioners. The most common Hawaiian healing practices were prayer, *pōpolo* (*Solanum nigrum*) berries, and *lomi* (body massage) to back and chest to help relax the child during an asthma attack and “on their nebulizer.” Other Hawaiian remedies included drinking warm water with salt, gargling water with *pa‘akai* (Hawaiian salt), *māmaki* (*Pipturus albidus*) tea, *‘aloe* (*Aloe vera*) tea, *noni* (*Morinda citrifolia*) juice, *‘ōlena* (*Curcuma longa*) tea, eating honey, cleansing with *kukui* candlenut (*Aleurites moluccana*), and breathing the vapors of eucalyptus leaves in hot water.

The relevance of the ocean for healing was a common theme. As one mother explained, “Basically every time my daughter get sick, bring her to beach. That’s how I grew up. You’re sick. We got to go to the water. And it’s fun for the kids*…* Everything [mucus congestion] all comes out.” The healing properties of the ocean are accompanied by the sun, moist sea breeze, mist from pounding waves, and salty water.

Focus group participants from Kaua‘i spoke about “full-blooded” Hawaiian friends and relatives from the private island of Ni‘ihau who know about Hawaiian medicine. They reportedly pray in the Hawaiian language, apply healing touch, and “do rituals that calmed the child.”

Although a grandmother advised giving *māmaki* or ‘*aloe* tea to the affected child, the parent reported having difficulty finding the plants. Other concerns for this parent included Hawaiian remedies being distasteful, ineffective, unclean (from potential animal waste), and possibly unsafe for young children.

Most parents in the study were unfamiliar with traditional Hawaiian healing practices. One parent explained, “I’m not going lie [I learned]*…*nothing [about Hawaiian healing practices]*…*growing up everything was always*…*, the doctors*…* Never taught us you know, the Hawaiian way. In a way I wish we did.” Another parent added, “Not too much Hawaiian classes on this island. Not too much Hawaiians on this island.” A participant in another group explained the differences between ethnic groups and healing practices. “My Hawaiian side is on my dad’s side and they all passed away so that’s kind of hard, so I was raised by Portuguese side. They don’t do that kind of stuff.”

Despite being unfamiliar with traditional healing practices, parents often cited the “Hawaiian way” and the influence of the Hawaiian culture on the care of their child. Here is an exchange about the family. “‘*Ohana*. The number one rule in the Hawaiian heritage [is] your family always comes first.” “Always.” “And your family is always there for you.” Cultural values described in other focus groups included *aloha* (kindness), inclusion, respect for nature, being humble, remaining thankful for what one has, not expecting reciprocal gifting, *kōkua* (helping others), and friendliness.

Several focus groups discussed concerns about the long-term dependence and effects of medications. Many parents reportedly preferred comfort measures and natural remedies over medicines. One parent explained her actions and concerns about administering medications.

The first is giving them the pump. They give her that Prednisone, but it makes her body so shaky and she doesn’t like it and she’ll freak out… What am I suppose to do if it helps her…? It’s [asthma medication] the fastest thing. But… if there’s other ways, it’s better I would think. Pharmaceuticals aren’t the safest thing to take…it might be helping one problem and eventually it causes another problem.

Another related, “Now we are going to a naturopath to see if that helps*…* Changing his diet. Cleaning everything. Just trying to stay away from the medicine cause it’s making it worse.” Another parent with several children with asthma shared her thoughts about medicine.

Back in the day, there really wasn’t any doctors…or diseases. And the Hawaiians didn’t get sick at all until later on… So for me, instead of running for medicine I’m always trying to keep that in mind. Back then, there wasn’t doctors. What did they do? What could they do? And that’s how I try to teach my kids…that helps a lot because it keeps my mind open to other options. I’m always looking for home remedies or natural medicine that I can use for my kids. Well, that’s just me.

### Construct: Adaptation

Positive adaptation involved goal directed behaviors that promoted the acquisition of developmental milestones and engagement in normal physical activities. Prevention was a new feature of positive adaptation. General good health was promoted by providing healthy diets and physical exercise such as running and swimming “to build up his lungs.” The most popular protective stratagem was, “[Drinking]*…*lots and lots of Hawaiian water. [Drinking] Hawaiian water is the best thing.”

Parents reported how they protect their children from smoke (cigarette, barbecue) and animal dander; keep the house clean; and encourage children to wash hands to prevent minor colds that triggered lengthy exacerbations. They monitor air quality and on “a high vog day,” some parents do not allow their children to play outside. Several parents spoke about moving to cooler areas of the island and altering the home environment (removing carpets and fabric curtains) to minimize exposure to asthma triggers. A son’s room was redesigned to face the sun to become warmer and have the wind blow through the front door for better ventilation. Special equipment to clean and filter the air were purchased, and air conditioners were installed. One parent restricts her adolescent from going to the annual community fair because the dust consistently triggers an asthma exacerbation and emergency room visit.

Parents with many years of asthma management experience reported involvement with schools to include writing emergency action plans, communicating with school personnel, and instructing their child on what actions to take during an asthma attack. One parent reported on a new school policy, “the best experience” whereby students with physician verification are not penalized for being tardy or absent due to morning asthma treatment or lengthy exacerbation.

Parents related how they were transferring the responsibility of asthma management to their children through education about asthma warning signs and “not to take their illness for granted,” guidance on when to seek help, “how to communicate to other people that they’re having trouble,” reminders to avoid asthma triggers, and “make sure they have their medicines.” A father expressed the eventual goal for his son by saying, “I just hope that I can set him up that he can leave my house and handle his own asthma. Have his own medical plan.”

Young children reportedly cooperated with asthma care and were able to “put it [nebulizer] all together.” Many older children, by parental account are taking the initiative to prevent exposure to asthma triggers and adopting self-care behaviors. Two children reportedly advised their mothers to stop smoking. One daughter broke all her mother’s cigarettes. School age children recognized asthma symptoms, communicated effectively with the teacher, and administered their own medication. An older school age child initiates his own remedy by “jump in the shower” because he likes the steam and runs the hot water until there is none left. Here is an account of a teenager’s adoption of Hawaiian remedies after witnessing a relative’s death due to asthma.

— now being big, he took all this Hawaiian medicine classes so he does everything all natural. He doesn’t do none of his nebulizers, no inhalers…he drinks his tea before he goes to school…drinks tea when he comes home, drinks tea before he goes to bed…. ‘Uhaloa [Waltheria indicia] leaves and the root and smashes it and makes…some tea. Then he goes with…the dad… [to the forest] and gets the eucalyptus leaves when he can feel himself coming down [asthma]. He’ll…get the leaves and boil it and sit in the tub with it…—also grew some ‘olena at the house… When he needs it, …goes outside the door. Pull em’, clean em’, and then he…juices that. That thing is nasty. But, he’ll drink it…

Biopsychosocial adaptation challenges involved health care delivery systems. The financial impact of caring for a child with asthma was reported to be a “*pilikia*” (difficulty) for many participants, particularly with private insurance. “Doctor bills, hospital bills, ambulance plan” and paying for equipment like the nebulizer machine make caring for a child with asthma “harder.” One parent reported “paying $150 a month just for meds that don’t even last that long.” Additional *pilikia* related to having to wait 2 weeks to obtain “prior authorization” from health insurance for medication refills, waiting for a referral to a specialist, and being denied reimbursement for alternative care such as chiropractic and naturopathy.

Geographic barrier was identified as a significant concern for parents living on the islands. Families living in distant communities travel 20–50 miles by car to see a pediatrician or for emergency hospital care. Public bus schedules and routes on the islands are limited. The price of gas on smaller islands is double the cost of gas on O‘ahu. For specialized emergency medical care, children are transported via air ambulance to Honolulu, O‘ahu. Distances from each island to Honolulu by air range from 200 miles (Hawai‘i island) to 50 miles (Moloka‘i) (State of Hawaii Data Book, 2015).

Parents on Lāna‘i, Moloka‘i, and Hawai‘i discussed the lack of available specialists such as pediatricians, allergists, dermatologists, and ear-nose-throat (ENT) physicians. One parent verbalized this common opinion, “…there’s plenty of people on this island with asthma. But we have to fly off island just to go see a pulmonologist. If there was one on island, would be so much more convenient.” One child was taken “many times to the doctors and they finally fly us out to Honolulu…he got to see one specialist and then they diagnosed him with asthma.”

Coordinating the family to fly to Honolulu was described as a “whole juggling act.” An itemization of the financial cost and psychosocial stressors involved in taking a child to O‘ahu includes airfare for child and parent(s), car rental/taxi, hotel, lost of work time, paying for extra luggage to carry a nebulizer machine, and securing childcare for the other children. One parent reported that, “Your $15.00 [insurance] co-pay ends up $1100.00.” Here is an explanation of an emergency situation on a small island.

The doctor bills and emergency bills. Will they get Medevac out? If they do, then…where are you gonna stay? One parent can only fly with your child…it separates the family…If you don’t have an [air] ambulance plan, it’s $16,000…one way… to Honolulu….[Parent] fly out…paying $278.00 ticket at the counter…So…$18,000 [for] the…whole weekend…[to] rent a car and hotels…

The educational system presented difficulties for children particularly if the teacher or school health aide was unaware of the child’s condition or unfamiliar with the signs and symptoms of an asthma exacerbation. One parent expressed her concern. “If the teachers are not aware that a child has asthma*…* [the child has] to do everything that the regular kids do*…*it sends some kids that have asthma into an attack*…* ‘My kid can’t breathe. She’s ready to faint. And you’re still telling her that she has to run?’”

Biopsychosocial adaptation difficulties were experienced by all parents. They expressed concerns about possibly making wrong decisions regarding asthma care. Additionally, parents conveyed disappointment about the limitations asthma impose on their children and worry for their safety when outside of parental supervision. Caring for their children with asthma was described as “very hard,” stressful, tiring due to lack of sleep worrying and “listening for that cough,” and sometimes overwhelming.

## Discussion

Distinctive challenges related to geographic barriers contribute to the uncertainty experience for native Hawaiian parents living on rural islands. Aboriginal peoples (First Nations, Inuit, and Métis) of Canada (Watson et al., 2012), indigenous New Zealand Māori (Jones, Ingham, Cram, Dean, & Davies, 2013), and Aborginal and Torres Strait Islander people of Australia (Giarola et al., 2014) live in remote areas and encounter similar geographic barriers to hospitals and spirometry testing (Castleden et al., 2016), social and health services (Ely & Gorman, 2010), specialty medical services (Chang et al., 2000), and asthma education and support (Watson et al., 2012).

In spite of geographic challenges, literature reports successful intervention programs involving native partnerships that provide specialty services, asthma education, and support to distant indigenous communities. In First Nation health centers across rural Alberta, Telehealth conferences are successfully led by Aboriginal peer facilitators and health professionals (Stewart et al., 2015). The employment of respected Aboriginal leaders as indigenous health workers (IHW) are essential in the Indigenous Health Model for Aboriginal and Torres Strait Islanders (Valery et al., 2016). IHWs provide community-based asthma education (Chang, Taylor, Masters, Laifoo, & Brown, 2010) and organize regular non-indigenous pediatrician/respiratory specialist visits to rural areas (Paterson, Nayda, & Paterson, 2012). Caregivers value specialists who “come to them” in their communities, are culturally aware, and able to develop positive connections (Valery et al., 2016). They feel empowered because of the education and support they received from IHWs (Valery et al., 2016). The *Tu Kotahi Māori*, Asthma Trust, provides culturally appropriate asthma services to their community utilizing a holistic Māori model of health in New Zealand (Jones et al., 2013). A 2-day “asthma camp” held in rural Nova Scotia provided opportunites for Aboriginal families to network and engage in favorable cultural activities such as prayer, drum-making, drumming, singing, dancing, art work, relay games, etc. (Stewart et al., 2015). Parents requested continued opportunities to learn more about asthma in their own language, join support groups to feel less alone, and apply cultural practices in the program.

Uncertainty was a common identified experience for parents in both *Mālama* studies. The lack of asthma care information, unidentified triggers, and unpredictable effects of asthma triggers were strong antecedents to the uncertainty experience. Similar findings included the identification of the health care provider and extensive ‘*ohana* as strong structure providers, concurrent use of Western medications and CAM remedies, and unfamiliarity with Hawaiian healing practices. Participants in both studies preferred natural healing remedies but recognized the effectiveness of medication for the treatment of acute asthma attacks. Men living on the islands were actively involved in their child’s asthma management. Parents with more experience and older children were adept at managing the asthma condition in both studies. The differences between the findings of first *Mālama* study (Kealoha & Kataoka-Yahiro, 2017) and the continued study centered on the rural islands’ lack of medical specialists and informational resources and the extra cost of accessing resources off-island in terms of money, time, and family involvement.

### Limitation

The limitation of this study is that it cannot be generalized to native Hawaiian and non-native Hawaiian parents who have children with asthma. The area of residence or locale may affect the experience of parents. The research sample may also have been a self-selected group of parents interested in sharing their experiences.

## Conclusion

Rich interview data was gathered from 33 native Hawaiian parents who participated in nine separate focus groups on the islands of Hawai‘i, Kaua‘i, Maui, Moloka‘i, and Lāna‘i. Data were organized into all components of the UIT and agreed with the findings of the first Mālama study of focus groups on O‘ahu. Contextual influences including indigenous worldview, cultural values, history, and assimilation and acculturation factors affected native Hawaiian parents’ perceptions and experiences with conventional asthma care. Moreover, Hawaiian parents living on the islands outside of metropolitan O‘ahu reported geographic barriers that contributed to their uncertainty experience.

Nurses, a group with the largest professional health care membership, are encouraged to become more politically active and lobby the federal, state, and county governments to deliver comprehensive medical services and educational and supportive programs for families living on all islands. Futhermore, nurses are urged to make full use of their varied positions to more strongly support native Hawaiian parents caring for their children with asthma by being respectful and culturally sensitive. Nurses are available to assist Hawaiian parents and communities to develop asthma management programs based on the concepts of Hawaiian health, cultural values, involvement of ‘ohana, and application of CAM therapies as well as standard asthma management.

## Declaration of Conflicting Interests

The author(s) declared no potential conflicts of interest with respect to the research, authorship, and/or publication of this article.
